# Efficacy and safety of intravenous beta-blockers in acute atrial fibrillation and flutter is dependent on beta-1 selectivity: a systematic review and meta-analysis of randomised trials

**DOI:** 10.1007/s00392-023-02295-0

**Published:** 2023-09-01

**Authors:** Madeleine Perrett, Nisha Gohil, Otilia Tica, Karina V. Bunting, Dipak Kotecha

**Affiliations:** 1 Institute of Cardiovascular Sciences, University of Birmingham, Vincent Drive, Birmingham, B15 2TT UK; 2 Cardiology Department, Queen Elizabeth Hospital, University Hospitals Birmingham NHS Foundation Trust, Birmingham, UK

**Keywords:** Atrial fibrillation, Atrial flutter, Beta-blockers, Acute, Systematic review, Meta-analysis

## Abstract

**Background:**

Intravenous beta-blockers are commonly used to manage patients with acute atrial fibrillation (AF) and atrial flutter (AFl), but the choice of specific agent is often not evidence-based.

**Methods:**

A prospectively-registered systematic review and meta-analysis of randomised trials (PROSPERO: CRD42020204772) to compare the safety and efficacy of intravenous beta-blockers against alternative pharmacological agents.

**Results:**

Twelve trials comparing beta-blockers with diltiazem, digoxin, verapamil, anti-arrhythmic drugs and placebo were included, with variable risk of bias and 1152 participants. With high heterogeneity (I^2^ = 87%; p < 0.001), there was no difference in the primary outcomes of heart rate reduction (standardised mean difference − 0.65 beats/minute compared to control, 95% CI − 1.63 to 0.32; p = 0.19) or the proportion that achieved target heart rate (risk ratio [RR] 0.85, 95% CI 0.36–1.97; p = 0.70). Conventional selective beta-1 blockers were inferior for target heart rate reduction versus control (RR 0.33, 0.17–0.64; p < 0.001), whereas super-selective beta-1 blockers were superior (RR 1.98, 1.54–2.54; p < 0.001). There was no significant difference between beta-blockers and comparators for secondary outcomes of conversion to sinus rhythm (RR 1.15, 0.90–1.46; p = 0.28), hypotension (RR 1.85, 0.87–3.93; p = 0.11), bradycardia (RR 1.29, 0.25–6.82; p = 0.76) or adverse events leading to drug discontinuation (RR 1.03, 0.49–2.17; p = 0.93). The incidence of hypotension and bradycardia were greater with non-selective beta-blockers (p = 0.031 and p < 0.001).

**Conclusions:**

Across all intravenous beta-blockers, there was no difference with other medications for acute heart rate control in atrial fibrillation and flutter. Efficacy and safety may be improved by choosing beta-blockers with higher beta-1 selectivity.

**Graphical abstract:**

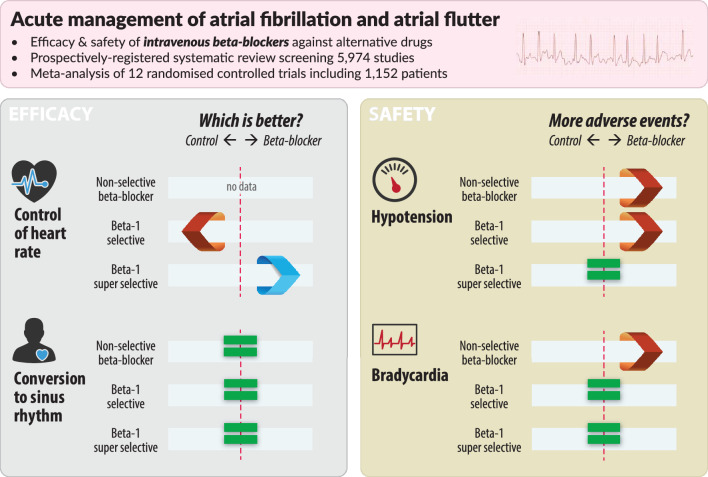

## Introduction

Atrial fibrillation (AF) and atrial flutter (AFL) are common forms of arrhythmia characterised by abnormal atrial activity, often accompanied by rapid ventricular response. The burden of AF as a proportion of the population is projected to dramatically increase year on year, and the high rates of morbidity and mortality pose a substantial burden on both individual patients and the healthcare system as a whole [1]. Guidelines for management suggest rate and rhythm control approaches, depending on haemodynamic stability, duration of onset and other clinical factors [2]. However even if rhythm control is instituted, most patients will initially be given rate control drugs while cardioversion is being considered. Beta-1 selective adrenergic blockers are usually the default option for management of atrial arrhythmias due to their wide application across cardiovascular medicine [3], however robust evaluation against other therapies is limited [4, 5].

In the context of acute AF/AFL management, intravenous therapy is often used to ensure rapid control of heart rate and facilitate early hospital discharge. A number of small trials have compared a variety of beta-blockers against other therapeutic agents. The conflicting results may be due to differences in the cardioselectivity and pharmacodynamics of the individual beta-blockers. A study of comparative effectiveness is critical in view of the frequent utilisation in routine care of intravenous beta-blockade, the availability of newer beta-blockers, and scant evidence to assist clinical decision-making. This systematic review aims to test the hypothesis that intravenous beta-blockers are superior, in terms of safety and efficacy, to other pharmacological interventions in the acute AF/AFL setting, whilst taking account of beta-1 selectivity.

## Methods

The systematic review was prospectively registered with the PROSPERO database (CRD42020204772). Ethical approval was not required as data were collected from published trials with pooling of anonymised results.

### Search strategy and eligibility criteria

MEDLINE, EMBASE and Cochrane Central Register of Controlled Trials (CENTRAL) were searched from inception to August 2020, using Boolean operators to group synonyms for relevant search terms. An example of the search terms used to build the search strategy is shown in Supplementary Table 1.

Study design was restricted to randomised controlled trials (RCTs), including parallel and cross-over designs. Studies were included if they investigated patients ≥ 18 years old with AF/AFL requiring acute treatment, and included at least one arm assessing intravenous beta-blocker therapy. Manual screening of relevant reviews and reference lists was also performed. Exclusion criteria included case reports, editorials, reviews, animal studies, and any studies published in abstract form or not available in English. Studies where intravenous beta-blockers were given in combination to other pharmacological agents, or as prophylactic therapy, were excluded. Studies which investigated supraventricular arrhythmias other than AF/AFL were also excluded.

A comprehensive search of title and abstract, followed by a full text screen was carried out by two independent reviewers (MP and NG), with adjudication of any discrepancies (KB).

### Outcomes

The primary outcomes were: (1) Reduction in heart rate after starting the randomised therapy, reported as a change from baseline in beats/minute or percentage decrease; and (2) Proportion of patients achieving study-defined heart rate control. Secondary outcomes were: (1) Rate of conversion to sinus rhythm, recorded as both number of patients who converted to sinus rhythm by the end of the study and, where applicable, mean time to convert to sinus rhythm; (2) Number of patients requiring electrical cardioversion; (3) Change in systolic blood pressure from baseline; (3) Number of adverse events, including hypotension, bradycardia, major cardiovascular events and major adverse events leading to drug discontinuation; (4) Time to hospital discharge; and (5) Mortality. Data on change in heart rate, change in systolic blood pressure and the number of patients who converted to sinus rhythm were extracted for the study-defined primary time point, as well as 30 min, 1, 2, 6, 12 and > 12 h post-treatment, where available. Definitions of adverse outcomes were accepted from each study; for hypotension, the majority of studies used a criteria of systolic blood pressure < 90mmHg, with one study also accepting a 20mmHg drop and one study < 80mmHg; for bradycardia, the majority of studies used a criterion of < 50 beats per minute (bpm), with one study < 60bpm.

### Data collection and risk of bias

Data on study design, patient demographics, drop-out rates and the aforementioned outcomes were extracted from each study using a pre-formulated spreadsheet. The Cochrane Collaboration tool was used to assess risk of bias across different domains (selection, outcomes, missing data, intervention, randomization and overall). As an example, biases relating to how heart rate outcomes were assessed, such as method (using electrocardiograms or clinical) and assessment (duration and/or timing) would be included under the measurement of outcomes domain. All data extraction and risk of bias assessment were performed by two independent reviewers (MP and NG), with adjudication of any discrepancies (KB).

### Data synthesis and statistical analysis

Baseline demographics including age, baseline heart rate and blood pressure were pooled with weighting for participants in each trial. For studies in which baseline values were not stated for each individual arm, missing data was imputed into each arm by using the overall value [6, 7].

Outcomes are described both qualitatively and quantitatively. Where sufficient data were available, meta-analysis was performed to compare beta-blocker and comparator groups. Beta-blockers were sub-grouped depending on their beta-1 selectivity: non-selective, beta-1 selective and beta-1 super selective. A random-effects model was used to meta-analyse across different beta-1 selectivity groups due to the anticipated variety in study designs and populations. A fixed-effects model was used to meta-analyse trials within each beta-1 selectivity group. Heterogeneity for each meta-analysis was assessed using the I^2^ statistic; a value over 50% indicates substantial heterogeneity across trials. Due to the large variety of time-point assessments for heart rate reduction, data were classified into ≤ 2 h or > 2 h; meta-analysis was only possible on data for heart rate ≤ 2 h. To compare numerical heart rate between groups, the standardised mean difference (SMD) was calculated, with associated 95% confidence intervals (CI). Where standard deviation was missing, this was imputed from the baseline value [8, 9]. The only trial with flecainide as a comparator [10] was excluded from the meta-analysis of conversion to sinus rhythm, due to its established role as an anti-arrhythmic agent. The differences in adverse events between beta-blocker and comparator groups were assessed with risk ratios (RR) and corresponding 95% CI. Statistical analysis was performed using Stata (version 14.2; StataCorp, Texas, USA). A 2-tailed p value of 0.05 was considered statistically significant.

## Results

**Fig. 1:**
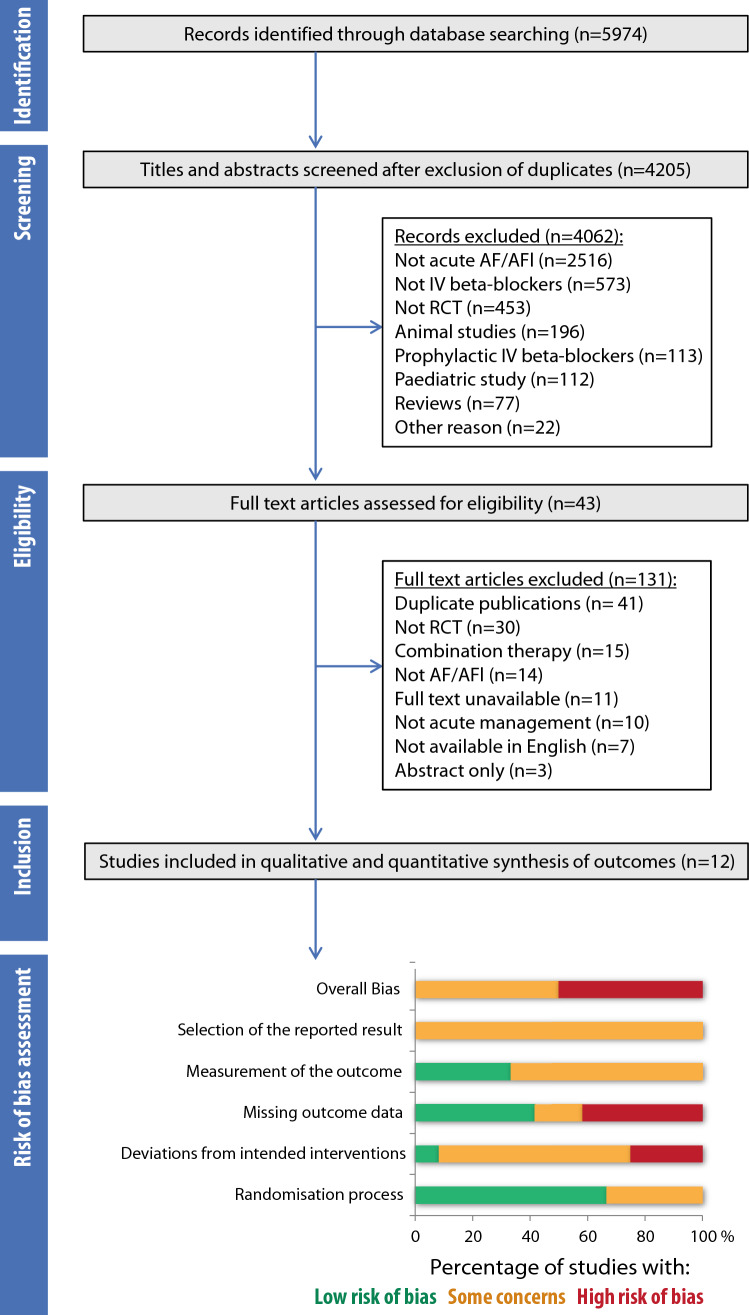
Study flowchart and risk of bias. *AF/AFL* atrial fibrillation/flutter, *IV BB* intravenous beta-blocker, *RCT* randomised controlled trial

The search strategy identified 5974 studies, of which 12 RCTs were included in the systematic review (Fig. 1) [6–17]. All 12 studies were conducted within secondary care, with 4 studies investigating post-surgical patients [10, 11, 14, 16]. Five trials were double-blind [6–8, 12, 17], two were single-blind [9, 10] and the remainder open-label (Supplementary Table 2).

Selection criteria varied across studies (Supplementary Table 3), with most AF/AFL patients only being included if they had a ventricular rate ≥ 100 bpm. The most common exclusion criteria were the presence of known obstructive lung pathology, or recent receipt of anti-arrhythmic medications. Risk of bias was variable in the included trials, with the highest level of bias in domains for missing outcome data and deviations from the intended intervention (Fig. 1 and Supplementary Table 4).

There were a total of 1152 adult patients across all studies, allocated to either intravenous beta-blocker therapy (n = 526) or a comparator (n = 626). Non-selective beta-blockers (sotalol, timolol) were used in 5 studies [6, 7, 10, 11, 17], selective beta-1 blockers (metoprolol, esmolol) in 5 studies [8, 12–15], and super-selective beta-1 blockers (landiolol) in 2 studies [9, 16]. Comparators varied, including diltiazem in 5 studies [8, 12–14, 16], digoxin in 2 studies [9, 11], placebo in 2 studies [6, 17] verapamil [15], flecainide [10] and ibutilide [7].

891 (78%) of the patients had AF and 123 (11%) patients had AFL, with some studies either not reporting AF/AFL individually, or reporting combinations of atrial arrhythmias [7, 9, 17]. Pooled weighted mean age was 62.4 years (SD 7.4), 38% were women and baseline heart rate was 137 beats/min (SD 11), with similar distribution in each randomised group (Table 1). As expected, there was a high rate of multi-morbidity (Supplementary Table 5).

**Table 1:** Pooled baseline characteristics

Characteristic	Beta-blocker (n = 526)	Comparator (n = 626)
Age; mean years (SD)	61.7 (7.9)	63.5 (7.8)
Women; n (%)	260 (39.2%)	286 (43.9%)
Systolic blood pressure; mmHg (SD)^a^	126 (9.0)	126 (8.1)
Diastolic blood pressure; mmHg (SD)^a^	79 (9.6)	81 (11.1)
Heart rate; beats/minute (SD)^a^	137 (11.9)	138 (10.5)
Hypertension; n (%)^b^	122 (55.2%)	125 (53.0%)
Diabetes; n (%)^c^	24 (22.6%)	27 (27.0%)
Long-term oral beta blocker; n (%)^c^	44 (27.0%)	53 (29.8%)
Long-term digoxin/digitalis; n (%)^b^	73 (24.7%)	76 (26.6%)

^a^Baseline blood pressure and heart rate are reported for 9 studies

^b^Hypertension and long-term digoxin/digitalis are reported for 5 studies

^c^Diabetes and long-term oral beta blocker are reported for 4 studies

### Primary outcomes of heart rate reduction

**Fig. 2:**
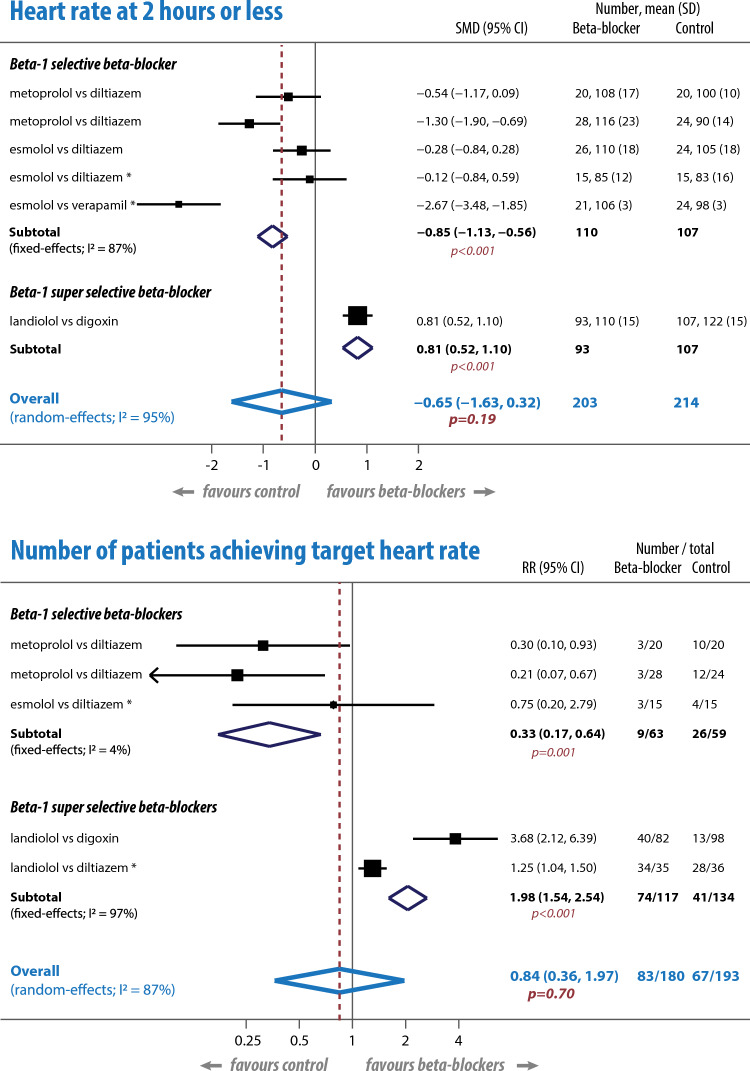
Meta-analysis of heart rate reduction. Crude heart rate reduction in beats/minute at ≤ 2 h (top panel) and number of patients achieving the study-specific target heart rate at the end of the study (lower panel). *Indicates a study where there are potential concerns about higher risks of overall bias. *CI* confidence interval, *I*^*2*^ heterogeneity across studies, *RR* risk ratio, *SD* standard deviation, *SMD* standardised mean difference

Six studies (419 participants) reported heart rate after intervention [8, 9, 12–15]. Combining time-points < 2 h, there was no difference in weighted mean reduction in heart rate between the beta-blocker (32 beats/minute) and comparator group (31 beats/minute). Meta-analysis showed substantial heterogeneity (I^2^ = 95%; p < 0.001) with no statistically significant difference between groups overall (SMD -0.65 beats/minute, 95% CI − 1.63 to 0.32); p = 0.19); Fig. 2. Analysis by beta-1 selectivity showed a distinct difference between the inferior results of five trials using conventional selective beta-1 blockers versus calcium channel blockers (SMD − 0.85 beats/minute, 95% CI − 1.13 to − 0.56; p < 0.001), compared to one trial of a super-selective beta-1 blocker against digoxin (SMD 0.81 beats/minute, 95% CI 0.52–1.10; p < 0.001).

In 5 studies (373 participants) [8, 9, 12, 14, 16] there was no statistically significant difference between beta-blocker and comparator arms in the proportion of patients achieving the study-defined target heart rate (RR 0.85, 95% CI 0.36–1.97; p = 0.70); Fig. 2 and Supplementary Table 6. Heterogeneity was substantial across all studies (I^2^ = 87; p < 0.001). There was no heterogeneity in sub-analysis of selective beta-1 blockers (I^2^ = 4%; p = 0.35), which were inferior to diltiazem in 3 studies [8, 12, 14] (RR 0.33, 95% CI 0.17–0.64; p < 0.001). In 2 studies against diltiazem and digoxin [9, 16], super-selective beta-1 blockers were superior (RR 1.98, 95% CI 1.54–2.54; p < 0.001).

### Conversion to sinus rhythm

**Fig. 3:**
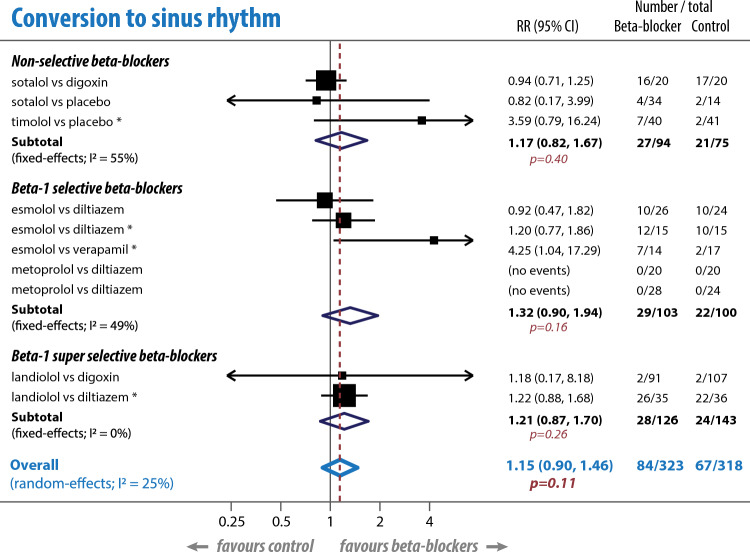
Meta-analysis of conversion to sinus rhythm. Number of participants who converted to sinus rhythm by the end of the study period (excluding one study using an anti-arrhythmic drug as a comparator). *Indicates a study where there are potential concerns about higher risks of overall bias. *CI* confidence interval, *I*^*2*^ heterogeneity across studies, *RR* risk ratio

Ten studies [6, 9–17] were included in the meta-analysis of the proportion of patients converting to sinus rhythm (641 participants). There was no statistically significant difference in the rate of conversion to sinus rhythm between beta-blocker and comparator groups (RR 1.15, 95% CI 0.90–1.46; p = 0.28), with no apparent effect of beta-1 selectivity and no heterogeneity; Fig. 3.

### Adverse events

**Fig. 4:**
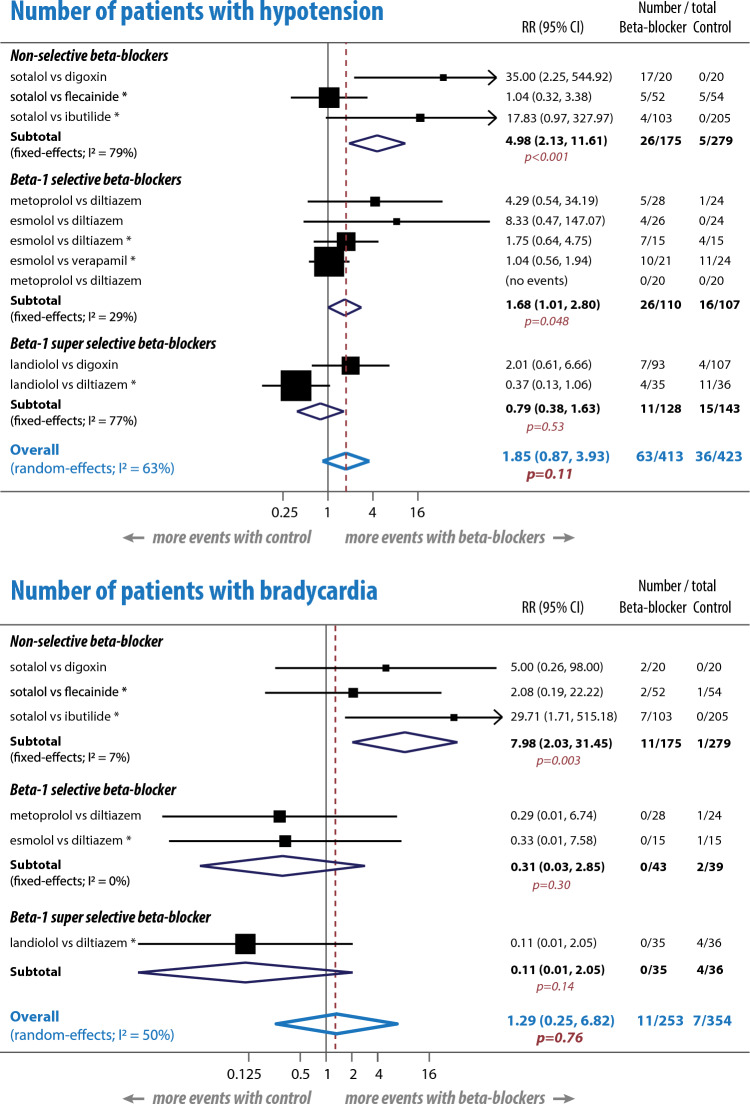
Meta-analysis of hypotension and bradycardia. Number of participants with incident hypotension (top panel) and bradycardia (lower panel). *Indicates a study where there are potential concerns about higher risks of overall bias. *CI* confidence interval, *I*^*2*^ heterogeneity across studies, *RR* risk ratio

Ten studies [7–16] (944 participants) reported adverse events, with hypotension and bradycardia having the highest incidence. Overall, there was no significant difference between beta-blockers and comparators in the incidence of hypotension (RR 1.85, 95% CI 0.87–3.93; p = 0.11), or bradycardia (RR 1.29, 95% CI 0.25–6.82; p = 0.76); Fig. 4.

Analysis by beta-1 selectivity identified significantly more hypotension events in the non-selective and beta-1 selective beta-blocker groups than comparators (RR 4.98, 95% CI 2.13–11.61; p < 0.001 and RR 1.68, 95% CI 1.01–2.80; p = 0.048), with no significant difference between super-selective beta-1 blockers and comparators (RR 0.79, 95% CI 0.38–1.63; p = 0.53). For bradycardia, there were significantly more events with non-selective beta-blockers than comparators (RR 7.98, 95% CI 2.03–31.45; p = 0.003), and no difference for either beta-1 selective or super-selective blockers. Adverse events leading to drug discontinuation were similar in beta-blocker and comparator groups (RR 1.03, 95% CI 0.49–2.17; p = 0.93), irrespective of beta-1 selectivity and with no heterogeneity between trials (Supplementary Fig. 1).

### Additional outcomes

Data were insufficient for meta-analysis of other outcomes. There were similar results for the beta-blocker and comparator groups for mean time to convert to sinus rhythm [7, 11, 13, 15], change in systolic blood pressure [8, 15], number of patients requiring electrical cardioversion [12, 14], major cardiovascular adverse events [7, 9, 11, 16] and time to hospital discharge [14] (Supplementary Table 7). No studies reported mortality rate.

## Discussion

Management of acute atrial fibrillation or flutter using intravenous beta-blockers resulted in no difference for heart rate control versus a range of comparator drugs, and no difference in adverse events such as hypotension or bradycardia. Clear distinctions were seen according to the degree of beta-1 selectivity, with super selective beta-blockers being more efficacious in terms of heart rate response, and non-selective beta-blockers being associated with more adverse events.

Acute atrial arrhythmias put a considerable strain on healthcare services and are a common reason for admission to hospital. The main goals when managing AF/AFL in the acute setting are to reduce the ventricular rate and promote conversion to sinus rhythm, whilst maintaining haemodynamic stability and minimising adverse events [2]. Preventing thromboembolic events is also critical, and as many patients will have other risk factors for stroke, commencement of anticoagulants is often advisable even for apparently ‘transient’ AF [18]. With spiralling healthcare costs, there is a clear need for rapid discharge of patients, and hence intravenous therapy is often instituted in the emergency department or acute admissions unit, often by general physicians. Although there has been extensive attention on rate versus rhythm control in the management of AF/AFL [19–21], in reality most patients are started on rate control therapy pending further assessment. Beta-blockers are the most commonly-used agents due to historical trial data across a range of cardiovascular conditions [22], but we lack robust analysis in AF/AFL that can inform the specific choice of beta-blocker versus other therapy. In chronic forms of AF, beta-blockers do not reduce mortality in patients with coexisting heart failure with reduced ejection fraction [22], and are not superior to alternatives such as digoxin [4]. However in the context of acute admission with AF/AFL, beta-blockers have a more clearly-defined role due to their speed of action, ease and familiarity of use. We hypothesised that the efficacy and safety of beta-blockers in the acute management of AF/AFL would be dependent on the selectivity against beta-1 adrenoreceptors. We tested this by performing a comprehensive systematic review and meta-analysis of RCTs to avoid the selection, information and confounding biases prominent in observational data.

In the management of acute AF/AFL, intravenous beta-blockers overall were no different to other agents used when considering heart rate reduction, achieving a target heart rate, or conversion to sinus rhythm. Beta-blocker therapy is often considered a ‘class’ drug in routine practice, but our findings clearly indicate that pharmacodynamic properties of the different beta-blockers have an impact on efficacy. With regards to safety, no statistically significant difference was seen comparing intravenous beta-blockers with alternative pharmacological therapy. This is reassuring for the use of intravenous beta-blockers in routine clinical practice. Similar to the efficacy analyses, we identified differences in adverse events according to beta-1 selectivity. Non-selective beta-blockers were associated with significantly more hypotension and bradycardia events than comparators (digoxin and anti-arrhythmic drugs), whereas beta-1 selective blockers were associated with more bradycardia compared to diltiazem. Super selective beta-1 blockers demonstrated better reduction in heart rate without any increase in hypertension, bradycardia or other adverse events. This balance of efficacy and safety would support more widespread use of highly selective agents in the routine management of acute AF/AFL. Although the number of studies using super selective beta-1 blockers was limited, and the comparison restricted to diltiazem and digoxin, the number of patients (and importantly events) was similar to the other beta-1 subgroups. Intravenous landiolol is around 8 times more beta-1 selective than esmolol, is ultra short-acting with a half-life of 4 min [23], and allows for a more rapid reduction in heart rate without a prolonged change in blood pressure [24, 25]. Super-selective beta-1 agents remain unavailable in many other countries despite evidence of cost-effectiveness in the European setting [26]. Our findings should encourage pharmaceutical companies to continue to develop additional selective beta-blocking agents, and to test these against a variety of other agents and across different clinical indications.

### Strengths and limitations

The design and outcomes for this systematic review were prospectively-registered, with screening, data extraction and risk of bias assessment independently performed by multiple evaluators. We were limited by the studies available for inclusion and the beta-blockers available in intravenous form, and hence could not examine common beta-blockers such as bisoprolol, or those with additional vasodilating properties such as nebivolol and carvedilol. Further, there were a wide range of comparator agents used across the included studies which took place in different clinical scenarios. This analysis was focused on comparative safety and effectiveness of beta-blockers with other agents, and did not compare beta-blockers against other beta-blockers. Exclusion criteria within the trials resulted in challenges for generalisability, most notably for pre-existing heart failure which is one of the commonest comorbidities in patients with atrial arrhythmias and associated with a considerable excess of adverse events [27, 28]. Significant heterogeneity was noted across the studies, with differences in dosage regimen and timeframe of intervention and comparator agents. However, this heterogeneity was substantially reduced by assessing within subgroups of beta-1 selectivity. Outcome assessment was variable (for example, the method used to evaluate heart rate change) and a number of outcomes relevant to routine practice were not presented, including time to hospital discharge and the number of patients requiring urgent electrical cardioversion. Risk of bias was variable, and overall assessments identified some concerns for six trials, and high risk of bias for the remainder six. Only two trials investigated super-selective beta-1 blockers (one with some concerns for risk of bias, and one with high risk). Further studies are clearly required in this clinically-important topic to cover all of these limitations, with careful attention to minimising risk of bias and improving generalisability to routine clinical care of patients with acute AF/AFL.

## Conclusion

Intravenous beta-blockers as a group were not superior to other pharmacological agents, but equally they were demonstrated as safe, even in the acute setting of AF/AFL. Significant differences were identified in the efficacy and safety of beta-blockers for the management of acute AF/AFL according to the degree of beta-1 adrenergic selectivity. More selective beta-blockers demonstrated a better balance of heart rate control without increasing adverse events such as hypotension and bradycardia.

## Data Availability

This study combined anonymous data from published articles; no additional data are available.
